# Emergence of ADC-5 Cephalosporinase in environmental *Acinetobacter baumannii* from a German tank milk with a novel Sequence Type

**DOI:** 10.1099/acmi.0.000485.v3

**Published:** 2023-06-26

**Authors:** Ellen M. E. Sykes, Valeria Mateo-Estrada, George Zhanel, Jeremy Dettman, Julie Chapados, Suzanne Gerdis, Ömer Akineden, Izhar I. U. Khan, Santiago Castillo-Ramírez, Ayush Kumar

**Affiliations:** ^1^​ Department of Microbiology, University of Manitoba Winnipeg, Winnipeg, Canada; ^2^​ Programa de Genómica Evolutiva, Centro de Ciencias Génomicas, Universidad Nacional Autónoma de México, Cuernavaca, Mexico; ^3^​ Department of Medical Microbiology and Infectious Diseases, University of Manitoba, Winnipeg, Canada; ^4^​ Ottawa Research and Development Centre (ORDC), Agriculture and Agri-Food Canada, Ottawa, Canada; ^5^​ Institute of Veterinary Food Science, University of Giessen, Giessen, Germany

**Keywords:** antibiotic resistance, beta-lactamase, food source, human health, pathogen

## Abstract

Bacteria resistant to antibiotics arguably pose the greatest threat to human health in the twenty-first century. One such bacterium that typifies antibiotic resistance is *Acinetobacter baumannii *. Frequently, hospital strains of *A. baumannii* display multidrug resistant (MDR) or extensively drug resistant (XDR) phenotypes, often requiring the use of last resort antibiotics for treatment. In addition to hospital settings, *A. baumannii* has been isolated from many highly divergent sources including wastewater treatment plant effluent, soil, and agricultural run-off with global distribution. However, such isolates remain poorly characterized. In this study, we characterized a strain of *A. baumannii,* AB341-IK15, isolated from bulk tank milk in Germany that demonstrated resistance to ceftazidime and intermediate resistance to ceftriaxone and piperacillin/tazobactam. Further genetic characterization identified an ADC-5 cephalosporinase, first incidence in an environmental isolate; and an OXA-408 oxacillinase that may contribute to this phenotype. Interestingly, AB341-IK15 is of a novel sequence type. This research underscores the importance of studying isolates of *A. baumannii* of non-clinical origin to understand the antibiotic resistance and virulence potential of environmental isolates of *A. baumannii* as well to understand the diversity of this species.

## Data Summary

Data for strain AB341-IK15 has been deposited in the NCBI’s Genbank repository under the biosample SAMN26898556, accession number JANBMU000000000.

**Impact Statement**

*
Acinetobacter baumannii* is one of the most important nosocomial pathogens that infects individuals with a weakened immune system and is difficult to treat with current antibiotics. However, not much is known about strains prevalent in various environmental niches. In this study, we have isolated a strain from tank milk that harbours an antibiotic resistance gene that confers resistance to a particular antibiotic class; the β-lactams. This is the first time this gene has been found in an environmental isolate of *A. baumannii *. Furthermore, we show that this isolate represents a novel sequence type that has never been seen before, implying that this environmental strain is different from hospital strains. These findings show that environmental strains are significantly diverse and harbour antibiotic resistance genes which could impact treatment success of *A. baumannii* infection in the hospital.

## Introduction

*Acinetobacter baumannii* is the World Health Organization’s top priority organism for which new antibiotics are critically needed [1]. Understanding ways by which *A. baumannii* acquires resistance to antibiotics are critical for the design of effective therapeutic interventions. *A. baumannii* has been historically isolated from hospital settings and much work has been done in clinical strains [2–6]. Up to 70 % of strains are multi-drug resistant (MDR) with many resistant to last resort antibiotics such as colistin and carbapenems [7]. Further, *A. baumannii* possesses various virulence factors such as biofilm formation, motility, secretion of proteases and iron acquisition systems that allow it to persist and thrive in a nutrient poor host environment leading to its success as a pathogen [8]. While clinical isolates of *A. baumannii* have been described extensively, there are limited studies characterizing environmental isolates of *A. baumannii *. However, recently it was suggested that this species needs to be considered a One Health problem, as several isolates from animals and plants belong to novel Sequence Types (ST) and have clinically relevant antibiotic resistance genes [9]. Thus, the study of environmental isolates of *A. baumannii* is necessary to identify the reservoirs of antibiotic resistance determinants and even novel lineages in this species. In this study, we characterize an environmental isolate of *A. baumannii *, AB341-IK15, that was isolated from bulk tank milk in Alsfeld, Germany using *Acinetobacter* spp. selective CHROMagar (CHROMagar, Paris, France) at 37 °C. It is not uncommon to isolate *Acinetobacter* spp. from dairy processing environments, as it is one of the top 25 most abundant and prevalent genera in pasture and feed, farm environments, teat skin, teat and bulk tank milk [10]. Accumulation of large numbers of antibiotic resistance genes (ARGs) are typical of *A. baumannii* isolates [11] and are of great concern as many are resistant to last resort antibiotics such as carbapenems and colistin [1]. Environmental isolates are also known to harbour such resistance mechanisms and may act as ARG reservoirs [12, 13]. Therefore, the purpose of this study was to characterize AB341-IK15 to identify determinants of antibiotic resistance and virulence present in this strain.

## Identification of *Acinetobacter* spp*.*

**Fig. 1.**
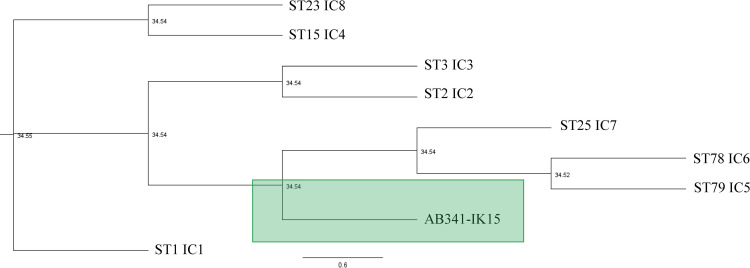
Phylogenetic relationship between Sequence Types (International Clones- IC) and AB341-IK15. Representative assemblies from each IC were used for this analysis and are as follows: IC1- SAMN07257378, IC2 – SAMN09667773, IC3- SAMN01828181, IC4 – SAMN09951355, IC5 – SAMN09951336, IC6 – SAMN12509149, IC7 – SAMN09951357, IC8 – SAMN03069270. The Pasteur Multi-locus Sequence Type (MLST) profiles of each IC were obtained, as well as the profile for AB341-IK15 using the PubMLST online webtool. The sequences for these seven genes were aligned using Clustal W and then concatenated using Geneious Prime. RAxML generated the phylogenetic tree. AB341-IK15 is highlighted in the green box and is in closest relation to ICs 5, 6 and 7 although is found in a clade all of its own.

Species misidentification is prominent within the *Acinetobacter* genus due to the large versatility and diversity within a single species combined with the fact that there is no simple technique for accurate identification. Phylogenetic markers and Average Nucleotide Identity (ANI) have been suggested as a more accurate way to identify *Acinetobacter* spp. [14]. Thereby, it is best to use a multi-pronged approach for identification and genomic characterisation of new *A. baumannii* isolates. For genotypic characterisation, a DNAeasy UltraClean microbial kit (Qiagen, MD, USA) was used to extract genomic DNA from a purified colony of AB341-IK15 according to the manufacturer’s instructions. Sequence libraries were prepared and pooled using the DNA prep and the NextSeq 500 mid output reagent kits (Illumina, CA, USA). Illumina NextSeq 500 platform, at the AAFC-ORDC, was used for whole-genome sequencing and *de novo* assembled using SPAdes v. 3.12.0 [15]. Quality assessments were performed using QUAST v 5.0.2 [16] and CheckM v1.0.11 [17] with a 95 % completeness and equal or less than 5 % contamination accepted, with AB341-IK15 meeting these criteria. The sequence has been deposited to the NCBI Genbank (biosample SAMN26898556 and accession JANBMU000000000). AB341-IK15 was identified as *A. baumannii* based on the ANI. To evaluate the relatedness of AB341-IK15 to other strains of *A. baumannii,* its sequence type (ST) was determined using the MLST pipeline [18] via the Pasteur scheme [19] which made use of PubMLST [20]. AB341-IK15 was found to be of a novel ST. The relationship of AB341-IK15 to each of the international clones (ICs) was evaluated using a phylogenomic approach. The allelic profiles of each IC as well as AB341-IK15 were established using the same method as AB341-IK15. After individual genes alignments were created using Clustal Omega 1.2.2 [21], sequences were concatenated using the index function in Geneious Prime and phylogenetic tree generated using RAxML v.8.2.11 [22] with a GAMMA model of rate heterogeneity and a maximum likelihood estimate of the alpha-parameter. As shown in Fig. 1, AB341-IK15 is most closely related to a group formed by IC4, IC5, and IC6, although it is on a different branch separate from all three of these. Characteristic profiles based on capsular polysaccharide (KL) type and lipopolysaccharide outer core (OCL) type are also actively used to track specific lineages of concern. Kaptive [23], a database originally developed for capsule typing for *Klebsiella pneumoniae *, has recently been supplemented with a database specific for *A. baumannii* [24]. Typically, this database uses a minimum threshold of ‘good’, meaning that the locus of interest was found in a single piece or with ≥95 % coverage, with ≤3 missing genes and ≤1 extra gene. These thresholds did not yield any results for AB341-IK15. However, using the loosest parameters, Kaptive determined that AB341-IK15 is within the KL95 and OCL22 lineages. Further work needs to be done with regards to the significance of these assignments as AB341-IK15 may represent novel alleles of these KL and OCL types. This exemplifies the diversity of *A. baumannii* and not only the emergence of novel lineages within the clinical setting [25] but also in the environment.

## Identification of ADC-5 Cephalosporinase

AB341-IK15 was tested for susceptibility to antibiotics. Using the Clinical Laboratory Standards Institute (CLSI) [26] broth microdilution method, the CANWARD panel of antibiotics were tested [27]. AB341-IK15 is susceptible to most antibiotics (Table 1). However, using CLSI breakpoints, it displays resistance to ceftazidime and intermediate resistance to ceftriaxone as well as to piperacillin/tazobactam.

**Table 1.** Antibiotic susceptibility of AB341-IK15. Susceptibility testing results for the CANWARD panel of antibiotics according to the Clinical Laboratory Standards Institute (CLSI) broth microdilution guidelines. AB341-IK15 is resistant to CAZ as indicated in bold and has an intermediate susceptibility to CRO and TZP as indicated in the underlined text. All values that indicate resistance are highlighted in bold text and those that indicate intermediate resistance are underlined. Data is displayed based on three biological replicates

Strain	AMK	CFZ	FEP	FOX	CAZ	BPR	C/T	CRO	CIP	CLR	CLI	CST	DAP	DOX	ETP	GEN	IPM	LZD	MEM	NIT	TZP	TOB	SXT	VAN
ATCC17978	1	>128	4	>32	8	0.5	2	16	0.25	32	>8	2	>16	0.25	4	1	0.25	>16	0.5	>512	4	1	4	>32
AB030	**64**	**>**128	**>64**	>32	**>32**	**>**32	32	**>64**	**>16**	>32	>8	0.5	**>**16	**32**	**>**32	**>32**	**>32**	>16	**>32**	>512	**>512**	**64**	>8	>32
AB341-IK15	2	>128	4	>32	**32**	0.5	4	16	0.25	32	>8	1	>16	1	4	≤0.5	0.5	>16	0.5	512	32	≤0.5	≤0.12	>32

AMK, Amikacin; BPR, Ceftobiprole; CAZ, Ceftazidime; CFZ, Cefazolin; CIP, Ciprofloxacin; CLI, Clindamycin; CLR, Clarithromycin; CRO, Ceftriaxone; CST, Colistin; C/T, Ceftolazane/tazobactam; DAP, Daptomycin; DOX, Doxycycline; ETP, Ertapenem; FEP, Cefepime; FOX, Cefoxitin; GEN, Gentamicin; IPM, Imipenem; LZD, Linezolid; MEM, Meropenem; NIT, Nitrofurantoin; SXT, Trimethoprim/Sulphamethoxazole; TOB, Tobramycin; TZP, Piperacillin/Tazobactam; VAN, Vancomycin.

**Fig. 2.**
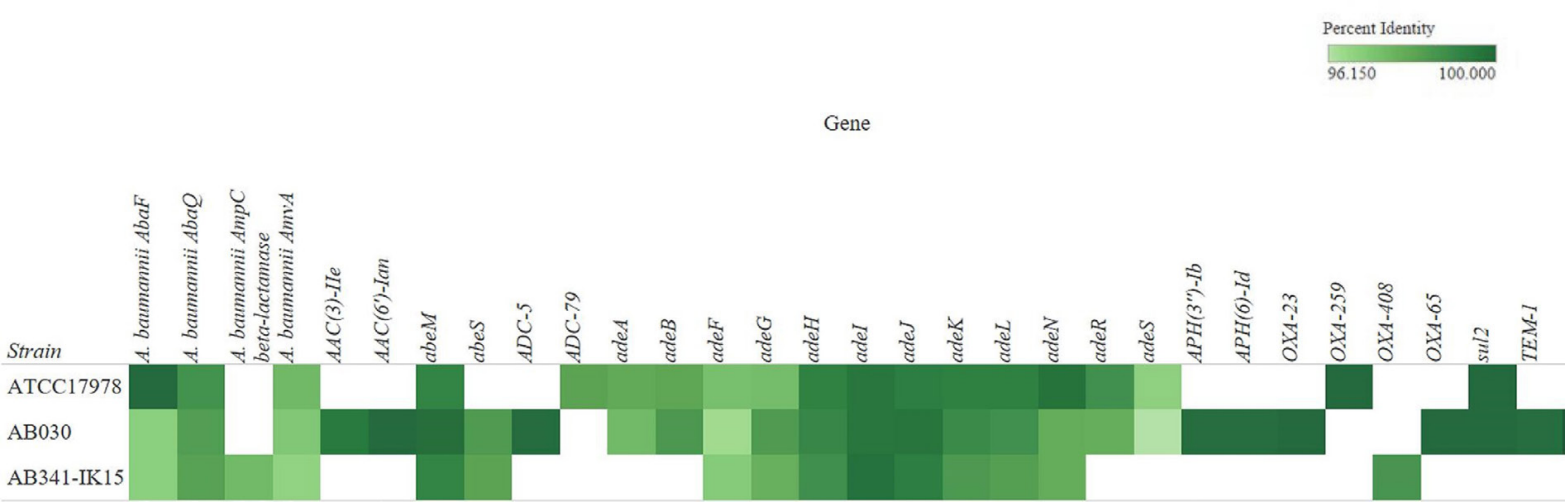
Percent identity of antibiotic resistance genes in AB341-IK15 and ATCC17978. Antibiotic resistance genes (ARGs) as determined using the Resistance Gene Identifier (RGI) in the Comprehensive Antibiotic Resistance Database (CARD) from February 2022. Only those hits matching the strict (95 %–99 %) and perfect (>99 %) criteria are shown. Note that there are two copies of OXA-23 encoded in AB030, both with 100 % identity but with only one copy represented. Differences between ATCC17978 and AB341-IK15 include the presence of ADC-5 and OXA-408 in AB341-IK15 as well as a lack of *adeAB, adeRS*, OXA-259 and *sul2* compared to ATCC17978. Comparison with AB030 yields greater differences, but with the common presence of *abeS*. Upon further investigation, the *A. baumannii* AmpC beta-lactamase only observed in AB341-IK15 was determined to be an ADC-5 cephalosporinase based on sequence identity.

**Fig. 3.**
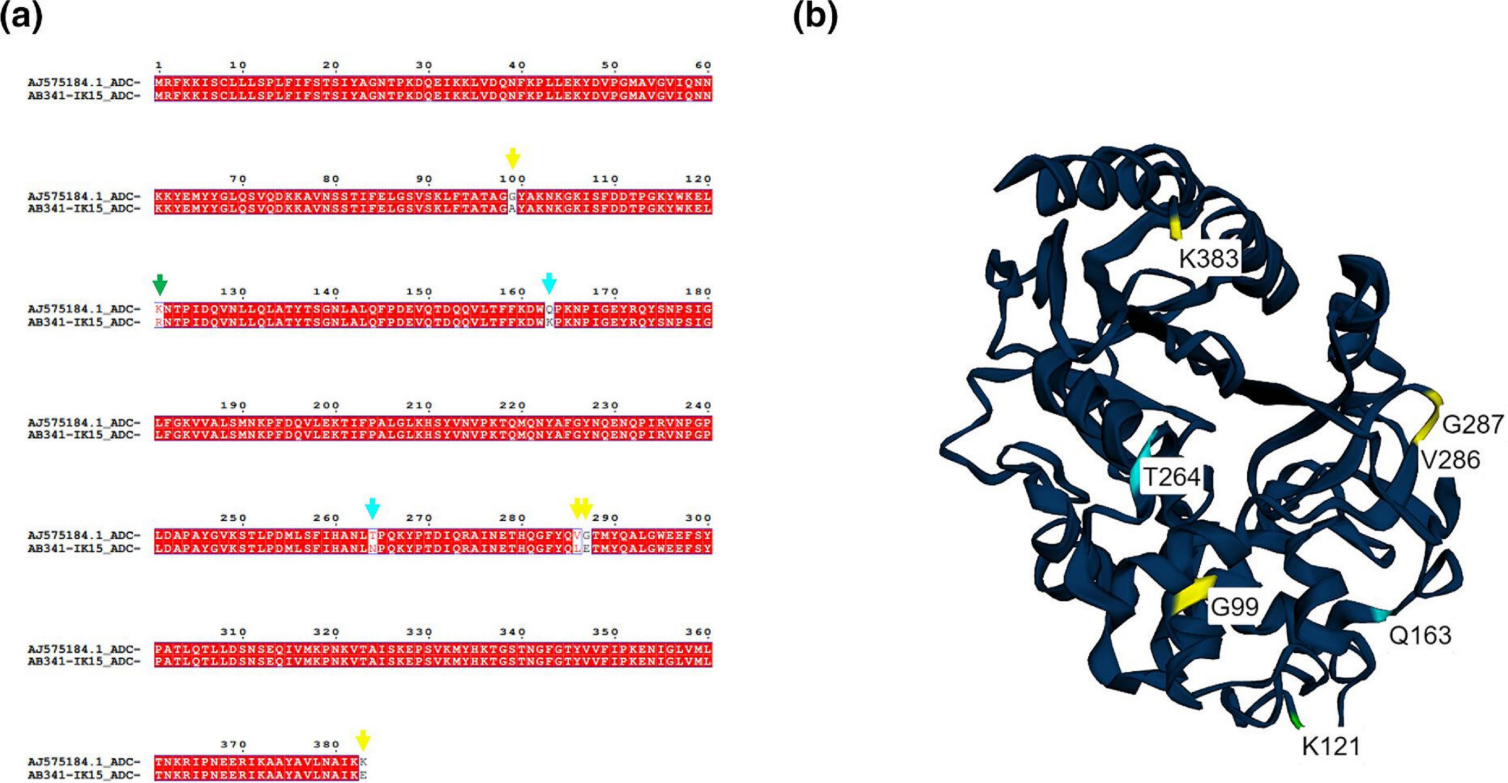
Nucleotide sequence of ADC-5 and its predicted structure. Alignment of putative ADC-5 cephalosporinase from AB341-IK15 and characterized ADC-5 AJ575184 (**a**). Structural predictions were done after amino acid alignment in MAFFT with ESPript. Characterized mutations are denoted with cyan arrows while novel mutations are highlighted with yellow arrows and K121R is highlighted with a green arrow as it is located in the binding pocket of ADC-5. Structural prediction of putative ADC-5_AB341-IK15_ using previously described ADC-5_AJ575184_, mutations in ADC-5_AB341-IK15_ are highlighted (**b**). Characterized mutations are highlighted in cyan while novel mutations are in yellow and green. G99A is located in the H2 α helices and K121R is a residue that makes up the binding pocket (highlighted in green).

In addition to susceptibility testing, further bioinformatic investigation analysed the AB341-IK15 scaffold using the Resistance Gene Identifier (RGI) in the Comprehensive Antibiotic Resistance Database (CARD) [28], via the ABRicate pipeline [29] in October 2022 considering perfect, and strict hits. The observed resistant phenotype to ceftazidime and intermediate resistance to ceftriaxone and piperacillin/tazobactam may be explained by the presence of an AmpC β-lactamase without carbapenemase activity, sharing 96 % identity to *bla_ADC-5_* (Genbank accession AJ575184) as well as a β-lactamase of 98.6 % identity with *bla_OXA-408_* (Genbank accession KJ584917) (Fig. 2). ADC-5 is a chromosomally encoded cephalosporinase originally identified in a clinical strain of *A. pittii* [30] and has only been characterized in MDR clinical isolates [31]. To the best of our knowledge, this is the first report of ADC-5 in an environmental isolate of *A. baumannii *. The nucleotide sequence of AB341-IK15 ADC-5 was translated using the ExPASy translate tool [32], aligned with AJ575184 using MAFFT with the G-INSi iterative refinement method [33], and analysed using ESPript (https://espript.ibcp.fr) [34] (Fig. 3a). The mutations observed in AB341-IK15 ADC-5 namely, Q163K and T264N, have been previously characterized via expression in *E. coli* and these mutations individually, are linked with a decrease in susceptibility to ceftriaxone, among other cephalosporins [35]. These mutations may explain the intermediate resistance phenotype observed in AB341-IK15. Furthermore, G99A, K121R, V286L, G287E, and K383E appear to be novel mutations. Using Phyre2 [36], ADC-5 (Genbank accession AJ575184) was modelled and the structure processed in EZMol v2.1 [37] to highlight the mutations in ADC-5 from AB341-IK15. Based on this analysis it can be seen that G99A is located in the H2 α helix (Fig. 3b, in yellow) which is known to be critical for β-lactamase activity [38]. Whereas, K121R, shown in green, contributes to formation of the binding pocket [39]. The impact of these mutations requires further investigation.

## Investigation of OXA-408 oxacillinase

OXA-408 is an intrinsically encoded oxacillinase and falls into the largest family of β-lactamases, the OXA-51-like [40]. Only two members of the family have been biochemically characterized and have a role in carbapenem resistance [41]. Modelling studies predict that ceftazidime is the strongest binding substrate for OXA-51-like β-lactamases [42] suggesting that OXA-408 in AB341-IK15 likely contributes to the clinical resistance phenotype. OXA-408 was originally identified in *A. baumannii* from a dog rectum in Zgharta, Lebanon [40] and therefore it is not necessarily unique to non-clinical settings. The use of third and fourth generation cephalosporins in agricultural practice in Germany has decreased by 52.64 % from 2011 to 2018 but hesitancy in compliance has made any further decrease difficult [43]. The continued use of cephalosporins in agriculture provides a likely explanation of the emergence of intrinsic resistance mechanisms such as ADC-5 and OXA-408 in *A. baumannii* isolates like AB341-IK15.

Comparing the CARD profile of AB341-IK15 with the type strain ATCC17978 (Genbank Accession NZ_CP018664) [44], as well as the hypervirulent, XDR hospital strain AB030 (Genbank Accession NZ_CP009257) [4], the differences between these strains are apparent (Fig. 2). Differences in the susceptibility profiles of ATCC17978, AB030, and AB341-IK15 are shown in Table 1. Comparable to ATCC17978, AB341-IK15, is susceptible to most antibiotics but differs by the lack of *adeAB, adeRS* and *bla_OXA-259_* which are present in ATCC17978 (Fig. 2). The absence of homologues in AB341-IK15 was validated using a manual blastn and tBLASTn search [45] using the specific gene entry in the CARD as the query. Although the blastn results validated the absence of these genes, upon further investigation using tBLASTn, homologues based on amino acid sequences were found. The tBLASTn search resulted in a hit for AdeA with a percent identity of 84.38 % with 92 % coverage, AdeB with 87.49 % identity and 99 % coverage, AdeR showed an 80.42 % identity with 97 % coverage, AdeS demonstrated 65.72 % identity with a coverage of 97 % and OXA-259 with a 97.08 % identity with 100 % coverage. These results suggest that putative alleles of these genes exist and require further investigation. Additional genotypic validation was performed via RT-qPCR using the Purelink RNA Extraction and DNase treatment kits as well as the VILO cDNA synthesis kit (Invitrogen, Waltham, USA) and SYBR green master mix (Applied Biosciences, Waltham, USA) using the StepOnePlus qPCR thermal cycler (Applied Biosciences, Waltham, USA). Relative normalized expression was calculated using the Pfaffl method with 16S rRNA as the reference gene and ATCC17978 as the reference strain [46]. In AB341-IK15, there was no detectable expression of *adeB* corroborating the genetic analysis performed with the RGI in the CARD (data not shown). The overexpression of *adeABC* is associated with aminoglycoside resistance in clinical isolates [47] and our data supports the clinical relevance of AdeABC due to the lack of homologues of this efflux pump in AB341-IK15 as well as the high degree of susceptibility to aminoglycoside antibiotics (Table 1). In contrast, AB030 and AB341-IK15 both show presence of the small multidrug resistance (SMR) family efflux pump, *abeS*. This is the only characterized member of the SMR family in *A. baumannii* and has been shown to play a minor role in susceptibility to chloramphenicol, fluoroquinolones, erythromycin and novobiocin [48]. Interestingly, AbeS pump was found to be upregulated when *A. baumannii* is exposed to colistin [49]. To what degree it plays a role in AB341-IK15 susceptibility needs to be studied further.

## Divergent *pilA* gene

**Fig. 4.**
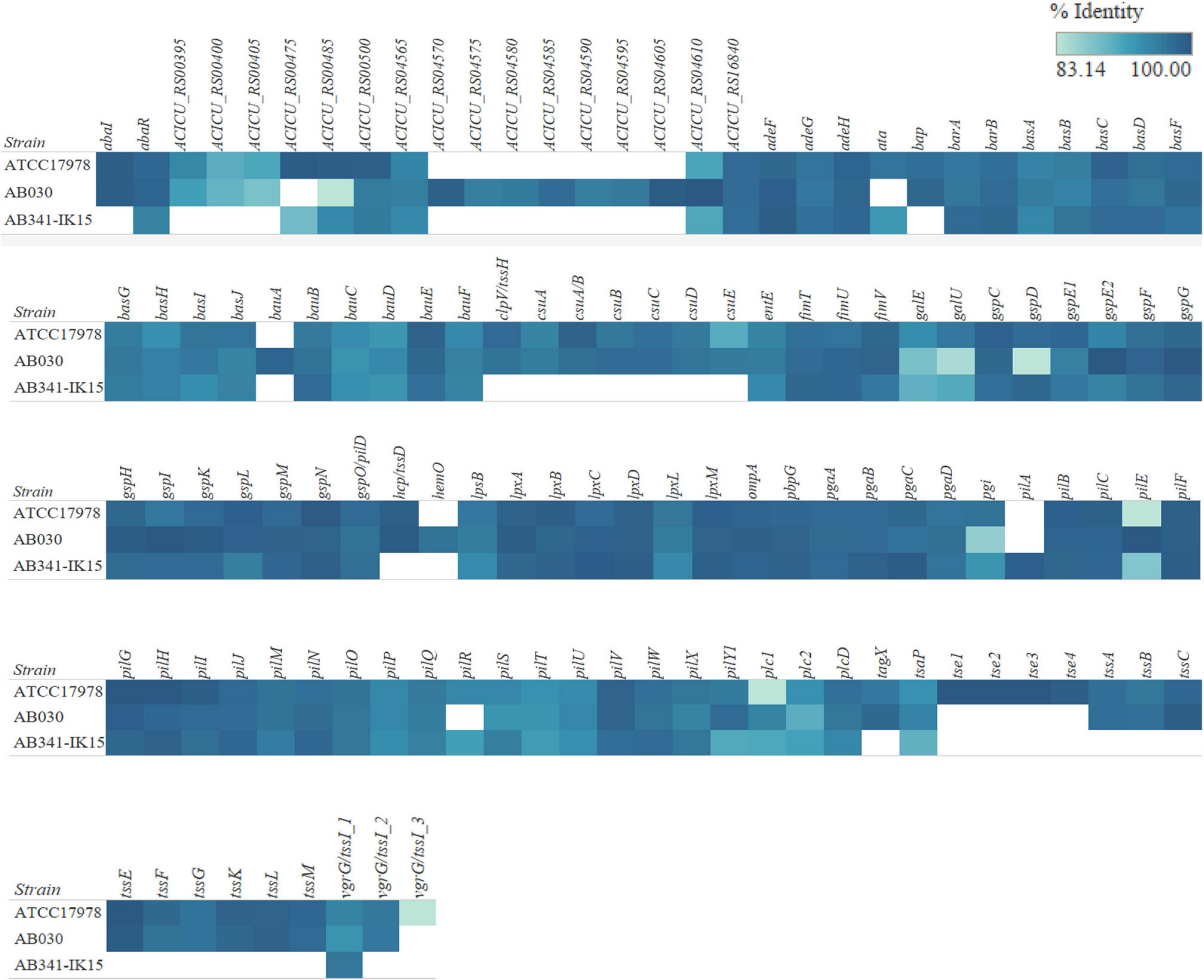
Virulence analysis of ATCC17978, AB030 and AB341-IK15. Analysis was carried out using the Virulence Finder Database in May 2022. All hits from the database are shown using a cutoff of 80% identity. The percent identity match is shown in gradient shades of blue with the darker colour highlighting a closer match to the database. If there is more than one copy of each gene, these are signified by a numerical value after the gene name indicating the copy number. AB341-IK15 contains a homologue of pilA involved in motility and biofilm formation in *A. baumannii *.

**Fig. 5.**
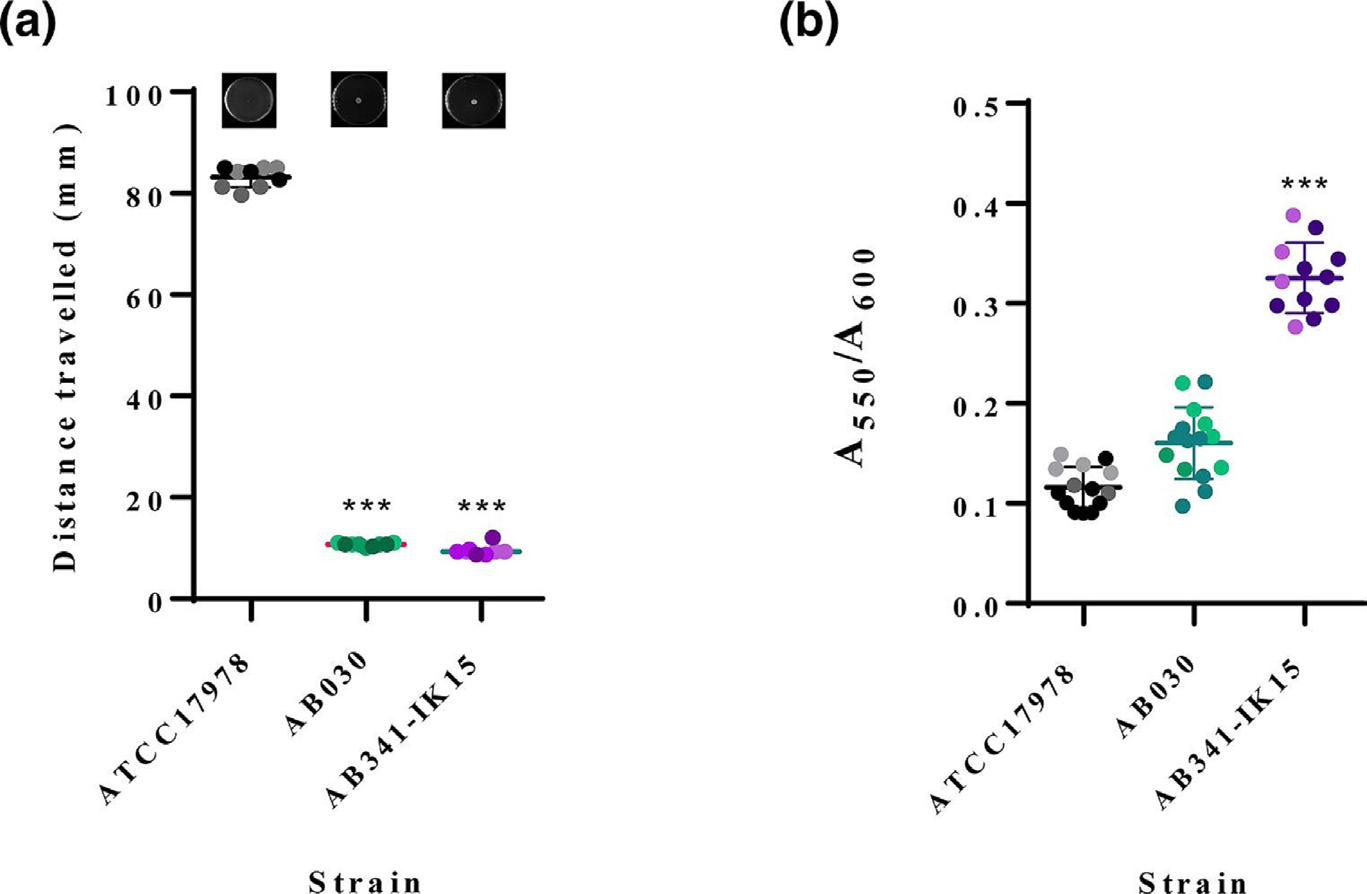
Motility and biofilm formation by ATCC17978, AB341-IK15 and AB030. ATCC17978 is a hyper-motile strain while AB341-IK15 and AB030 are non-motile (**a**). Motility experiments were performed with three technical replicates for each of three biological replicates. Biofilm formation quantification of ATCC17978, AB030 and AB341-IK15 (**b**). AB341-IK15 produces more biofilm than the type-strain ATCC17978 and the MDR clinical isolate AB030 as determined by the crystal violet staining assay. The data has been normalized to the OD_600_ of each strain to account for differences in cell density within the biofilm. Data was generated with five technical replicates for each of three biological replicates. Statistical analysis was performed in GraphPad Prism 9.3.1 using a one-way ANOVA.

In addition to ARGs, *A. baumannii* employs a multitude of virulence mechanisms including biofilm formation, motility, and protease secretion which provide advantages for survival in harsh conditions as well as persistence during an infection [8]. To investigate the virulence potential of AB341-IK15, again via the ABRicate pipeline accessed in May of 2022 (Seeman n.d.) the Virulence Finder Database (VFDB) [50] was used to classify such putative genes using a threshold of 80 % identity and 80 % coverage. These can be observed in Fig. 4. Immediately obvious is the presence of a gene homologous to *pilA* in AB341-IK15. Upon further validation via blastn, the AB341-IK15 putative *pilA* gene has 99.32 % identity to the gene found in *A. baumannii* ACICU (ACICU_RS16915, Genbank Accession CP000863) while ATCC17978 and AB030 have only 23 and 22% coverage, respectively, compared to ACICU, supporting the fact that these *pilA* genes are highly divergent from that of ACICU. PilA is a part of the type IV pili (T4P) assembly in *A. baumannii* [8]. T4P is involved in virulence phenotypes such as motility [51], natural transformation [52] and biofilm formation [53]. In *A. baumannii *, PilC is a platform protein that interacts tightly with the extension ATPase, PilB, and the retraction ATPase, PilT. PilA is the major pilin subunit, and upon assembly with other PilA subunits, forms the functional pilus. The *pilA* gene shows high divergence within the species [8, 53]. Comparison of *pilA* from clinical isolates ACICU, AB5075 and BIDMC57 demonstrated that glycosylation and other biochemical differences resulted in an inverse relationship between biofilm formation and motility [53] as observed in *Pseudomonas aeruginosa* [54]. This led to an investigation into the motility and biofilm formation capabilities of AB341-IK15. Using minimal motility media with 0.3 % agarose, overnight cultures of AB341-IK15, ATCC17978 and AB030 were normalized to an A_600_ of 1.0 and then 3 µl of the culture was stab inoculated into the centre of the plates. After incubation at 37 °C for 18 h, the diameter of the distance travelled was measured across three locations, and then averaged [55]. An ordinary one-way ANOVA test was applied for statistical analysis using GraphPad Prism v.9.3.1. Interestingly, in comparison to ATCC17978, a hyper-motile strain, AB341-IK15 appears to be non-motile under the conditions tested (Fig. 5a). Previous studies have shown the induction of motility in non-motile strains under conditions without stressor molecules such as sodium chloride [56], so it is possible that AB341-IK15 is motile under such other conditions as well but this needs to be investigated further. Biofilm formation was evaluated using a modified protocol [55], 96 well flat bottom plates were inoculated with 150 µl cultures standardized to A_600_=0.005 and incubated at 37 °C for 48 h. After which, planktonic cells were removed via washing with mQH_2_O and then the biofilm was stained with 0.1 % crystal violet for 30 min. Removal of the stain, followed by dissolution with 30 % acetic acid then allows for the measurement of the solubilized biofilm at A_550_. AB341-IK15 exemplifies this inverse relationship between biofilm formation and motility, being non-motile (Fig. 5a) while forming quantitatively more biofilm than either ATCC17978 or AB030 (Fig. 5b). Considering AB341-IK15 *pilA* is highly similar to ACICU *pilA*, the virulence potential of AB341-IK15 could be considerable. ACICU is predicted to participate in trans-bundling of its pili with other cells in the vicinity and is thereby able to better form microcolonies leading to an increased ability to form biofilms [53] and this may in fact be the case with AB341-IK15. Further investigation into *pilA* in AB341-IK15 is ongoing.

## Summary

In summary, evaluation of the ARG and susceptibility profiles of environmental isolates is vital to better understand the resistance potential of *A. baumannii *. AB341-IK15 represents an isolate of novel ST supporting the fact that the diversity of the species continues to expand. This isolate serves as an example where non-clinical isolates of *A. baumannii* not only harbour antibiotic resistance gene(s) but also display resistance to antibiotics. Thus, our work contributes towards the knowledge base to fully understand the diversity of *A. baumannii *. Notably, this is the first time the cephalosporinase ADC-5 has been identified in an environmental *A. baumannii* isolate. The mutations in AB341-IK15 ADC-5 are consistent with those in the literature suggesting a decrease in susceptibility to ceftriaxone, which corresponds to what is observed phenotypically. Two additional mutations in the AB341-IK15 ADC-5 are novel and their contribution to susceptibility will be investigated in future studies. In conclusion, our study underscores the importance of studying non-clinical *A. baumannii* isolates for a better understanding of the reservoirs of resistance and virulence determinants in *A. baumannii *.
